# Knowledge and awareness about and use of iodised salt among students in Germany and Greece

**DOI:** 10.1186/s12889-022-14008-9

**Published:** 2022-10-04

**Authors:** Katharina Heimberg, Annett Martin, Anke Ehlers, Anke Weißenborn, Karen Ildico Hirsch-Ernst, Cornelia Weikert, Britta Nagl, Antonios Katsioulis, Lamprini Kontopoulou, Georgios Marakis

**Affiliations:** 1 German Federal Institute for Risk Assessment, Max-Dohrn-Str. 8-10, 10589 Berlin, Germany; 2 University of Thessaly, 41500 Gaiopolis, Greece; 3 Hellenic Food Authority, Kifisias Ave. 124 & Iatridou St. 2, 11526 Athens, Greece

**Keywords:** Iodine, Iodised salt, Iodine food sources, Knowledge, Awareness, Public health, Survey, Germany, Greece

## Abstract

**Background:**

Iodine is an essential trace element, which is important for human metabolism, growth and mental development. Iodine deficiency may still occur in Europe and the use of iodised salt is an effective measure to enhance iodine intake. Knowledge and awareness about the importance of iodine in nutrition and health can have a positive impact on the use of iodised salt. Therefore, the aim of this study was to assess the knowledge about and use of iodised salt among university students in two European countries.

**Method:**

Data from two countries (Germany and Greece) were extracted from a multi-centre cross-sectional survey, conducted among non-nutrition science/non-medical students from October 2018 to April 2019.

**Results:**

Among the 359 participants in Germany (35% females, median age: 22 years) and the 403 participants in Greece (51% females, median age: 21 years), 41% and 37%, respectively, reported use of iodised salt at home. Users and non-users did not differ by age, gender and Body Mass Index or general interest in nutrition in both cohorts. However, those who had a better knowledge about iodine and (iodised) salt or had previously attended nutrition classes were more likely to report iodised salt usage.

**Conclusion:**

The results suggest that strengthening the imparting of nutritional information and additional education of young adults are needed and may improve knowledge about and usage of iodised salt.

## Key Points

- The degree of iodised salt use among university students in Germany and Greece was low with 41% and 37%, respectively
- Vegans (German study sample) had a higher iodised salt use level (57%) than students who followed other plant-based diets or were omnivores (36% and 47%, respectively)
- The overall observed knowledge about iodine and iodised salt in nutrition and health was unsatisfactory, but better in females, in both countries
- An increasing knowledge and the attendance of a nutrition course were associated with a more frequent use of iodised salt
- There is a need for raising awareness and knowledge about the importance of iodine for human health as well as about relevant iodine food sources and for increasing the use of iodised salt among young adults in Germany and Greece.

## Background

Iodine is an essential trace element with vital functions for human growth, metabolism and mental development, which are mediated by the synthesis of thyroid hormones. Long-term iodine deficiency is associated with an increased frequency of thyroid disorders [1, 2]. The consequences of especially severe intrauterine iodine deficiency have been known for a long time and include an increased risk of miscarriage and stillbirth, or even the most extreme form of iodine deficiency disease referred to as cretinism, although this is very rare nowadays [1]. However, even a mild to moderate iodine deficiency in utero and in early life-stages may have a negative impact on the child´s development with long-term health consequences. A mild to moderate iodine deficiency in adults may lead to adverse effects secondary to hypothyroidism, including impaired mental function with decreased educability and reduced work productivity [1, 2].

The European Food Safety Authority (EFSA) has set an adequate intake (AI) for iodine at 130 to 150 µg/day for adolescents from 15 years of age and adults and at 200 µg/day for pregnant and lactating women [3]. The main factor responsible for not achieving iodine sufficiency is an inadequate dietary intake. This mainly occurs in populations living in areas with a low iodine soil content [4], but it may also occur due to infrequent consumption of sea fish and a low use of iodised salt at the household level and, above all, by the food industry [1].

In order to ensure an adequate intake and prevent iodine deficiency disorders (IDD), salt fortification with iodine has been implemented as a prophylactic measure in both Germany and Greece, following official recommendations and based on the universal salt iodisation (USI) strategy by the WHO and UNICEF. However, in many countries of the world—including Germany and Greece—the addition of iodine to salt occurs on a voluntary basis and is thus far from being universal at present [1]. According to the latest national food consumption survey (Nationale Verzehrsstudie II; NVS II) that was conducted in Germany between 2005 and 2007, the main natural food sources contributing to iodine intake—in order of the most relevant sources—were milk and milk products, non-alcoholic beverages (water, coffee, tea, fruit juices, vegetable juices and lemonades because of the natural iodine content of the water) and sea fish. However, when being produced with iodised salt, processed foods such as meat, meat products and bread are the most relevant iodine sources [5]. There are no recent data available about the main food sources of iodine from Greece [6], but data from 1993 indicate that milk and milk products, meat and sea fish served as the most important contributors besides iodised salt [7].

Despite improved socioeconomic conditions and the voluntary implementation of salt iodisation in Germany and Greece, which ameliorated the population´s iodine status over the past decades [1, 8], recent data indicate a decreasing iodine status in German children and adults [9–11] and a suboptimal iodine status in pregnant women in Greece [12, 13].

A few international studies revealed a positive impact of good knowledge and awareness about iodine/iodised salt on the use of iodised salt at home and on iodine intake [14–16], and a low level of knowledge has been suggested to be a risk factor for suboptimal iodine intake or iodine deficiency [17–19]. We recently published results about knowledge, awareness and behaviour regarding salt and iodine among university students in European and Asian countries (Germany, Greece, Poland, Slovenia, Sri Lanka and Taiwan), with a major focus on salt use [20]. The objective of the present study is to assess knowledge and awareness about the importance of iodine/iodised salt for health and its use at home, based on data from the German and Greek subpopulations of the original multicentre study. We also aimed at determining whether the (conscious) use of iodised salt was associated with age, gender, Body Mass Index (BMI) as well as with knowledge regarding iodine and related factors such as attendance of a nutrition course, interest in nutrition and the habit of reading food labels.

## Methods

### Participants and questionnaire

Survey methods have been described in detail elsewhere [20]. Shortly, universities (of applied sciences) were selected randomly, informed about the objectives, design and methodology of the study and asked for permission to collect data from students in their premises. Trained research staff randomly selected departments/class-rooms of those universities that had agreed on participation and arranged times for data collection with the respective lecturers. Following a short introduction on the objective of the survey and information about the voluntary and anonymous participation, self-administered paper-based questionnaires were disseminated to the students. The questionnaire contained questions on students´ behaviour and knowledge/awareness regarding salt and iodine/iodised salt, since public health measures for optimising salt and iodine intake are closely linked [21], but also on the actual use of iodised salt for cooking and food preparation. Furthermore, it contained questions about participants’ sociodemographic data (age, gender and anthropometrics), their interest in nutrition, whether they had ever attended nutrition courses, about their habit of reading food labels, and—only in Germany—about their dietary habits, i.e. whether they followed an omnivorous, vegetarian, vegan or other types of plant-based diets (pescetarian, flexitarian).

The questionnaire was provided in the respective languages, thus participants had to be able to read and write these languages. Based on the assumption that nutrition and medical students might have a better knowledge about iodine, students from those faculties were not included in this survey. The study was approved by the responsible ethical review boards, i.e. the Ethics Committee of the Berlin Chamber of Physicians and the Ethics Committee of the Technological and Educational Institute of Thessaly.

### Assessment of knowledge about iodine/iodised salt

To rank the level of students´ knowledge about iodine/iodised salt, the nine knowledge-based questions of the questionnaire were selected and transformed into a knowledge score (Table 1). A question with only one possible correct answer was assigned one point for correct and zero points for wrong or uncertain (“I do not know”) response. For multiple-choice questions with more than one correct answer, one point was assigned for each correct answer ticked and each wrong answer correctly not ticked.

**Table 1:** Question items included in the knowledge score

Question items	Knowledge-based questions
	***Do you know the max. amount of salt that experts recommend to be consumed by adults per day?***
**Q1**	**5–6 g**^a^
	Or
	**1 Teaspoon**^a^
	***How much salt do you think children should consume compared to adults?***
	More salt than adults
	The same amount of salt as adults
**Q2**	**Less salt than adults**^a^
	I do not know
	***Do you know if a diet high in salt is related to any of the following diseases?***
**Q3**	**Kidney stones**^a^
**Q4**	**High blood pressure**^a^
**Q5**	**Osteoporosis**^a^
**Q6**	**Stomach cancer**^a^
**Q7**	Obesity
	***Do you believe that those who do sports (as a hobby and not as a profession) need more salt in their diet compared to those who do not do any sports?***
	Yes
**Q8**	**No**^a^
	I do not know
	***Which of the following is the most relevant source of salt in the diet of adults?***
	Salt added during cooking
	Ready-made sauce/stock cubes added during cooking
	Salt added on the plate
	Ready-made sauce (e.g. soya sauce etc.) added on the plate
**Q9**	**Salt in all types of processed foods (e.g. bread, cheese etc.)** ^a^
	Salt naturally occurring in foods
	***Do you think that Himalayan salt, as far as its iodine content is concerned, is a…?***
	Better source of iodine than iodised salt
**Q10**	**Poorer source of iodine than iodised salt**^a^
	Neither better nor worse
	I do not know—I have never heard of Himalayan salt
	***Are any of the food items below, in your opinion, good sources of iodine in the diet?***
**Q11**	**Fish**^a^
**Q12**	**Milk**^a^
**Q13**	**Seaweed**^a^
**Q14**	**Iodised salt**^a^
**Q15**	**Red Meat**^a^
**Q16**	Fruits
**Q17**	Nuts
**Q18**	Soy sauce
	***Do you know if any of the following population groups require additional iodine or are at risk of becoming deficient in iodine?***
**Q19**	**Pregnant women**^a^
**Q20**	**Lactating women**^a^
**Q21**	**Vegans**^a^
**Q22**	Athletes
**Q23**	Elderly people
**Q24**	Vegetarians
	***A diet low in iodine increases the risk for/ is related to…?***
**Q25**	**Poor cognitive development**^a^
**Q26**	**Thyroid disorders/disease**^a^
**Q27**	Obesity
**Q28**	High blood pressure
**Q29**	Skin rash

^a^correct answers contentwise

The total knowledge score ranged from 0–29 points and, due to the lack of a validated cut-off point for the iodine knowledge score used here, was divided into 3 categories: low knowledge (0–9 points), medium knowledge (10–19 points) and high knowledge (20–29 points).

### Statistical analyses

Data from 359 German and 403 Greek students in Germany (Berlin) and Greece (Larisa and Thessaloniki), collected in the original study [20], were used for the following analyses. Before statistical analysis, students that were under the age of 18 or above the age of 35 (*n* = 12) were excluded since these students did not represent our study group of young adults.

#### Univariate and multivariate analyses

In the univariate analyses, chi-square independence tests were performed and Odds Ratios (OR) with a 95% confidence interval (95% CI) calculated. Differences in continuous variables between groups, i.e. age, BMI and knowledge score, were analysed using the Mann–Whitney U-test. The effect size was calculated using the Z-statistics and the total sample size by use of the formula: $r=\frac{z}{\sqrt{N}}$. According to Cohen [22], an effect size of 0.1 to < 0.3 indicates a weak correlation and of 0.3 to < 0.5 or ≥ 0.5 a moderate or strong correlation, respectively.

For multivariate analyses, a logistic regression model was applied that included the variables age, gender, BMI, attendance of a nutrition course, interest in nutrition, food label use and the knowledge score. Effects of interaction were explored between the prior attendance of a nutrition course and the knowledge score. *P* values < 0.05 were considered as statistically significant.

#### Rasch modelling and Wald test

To further evaluate the knowledge of the study population, an item response analysis was conducted by use of the Rasch modelling approach, which is a statistical probabilistic model that requires the scoring of participants´ responses (based on the 29 single question items used here) into dichotomous variables, i.e. “1” for correct answers and “0” for incorrect answers, in analogy to the knowledge score.

As a result of the Rasch modelling, a non-linear Item Characteristic Curve (ICC) graphically describes the probability of a certain question item being answered correctly by a specific person, taking into account a person´s individual ability and the difficulty of the question items. The model equation for this analysis is the following:

$$P\left({X}_{vi}=1|{\theta }_{v},{\beta }_{i}\right)=\frac{\mathrm{exp}({\theta }_{v}-{\beta }_{i})}{1+\mathrm{exp}({\theta }_{v}-{\beta }_{i})}$$

$${\theta }_{v}$$ is the ability of a participant ($$v)$$, $${\beta }_{i}$$ is the difficulty of the item (*i*)$$,$$ and $$P\left({X}_{vi}=1|{\theta }_{v},{\beta }_{i}\right)$$ is the probability that participant $$v$$ gives a correct answer to item $$i$$.

The exponential function $exp$ is based on the Euler number *e* (2.71828…) and determines the course of the respective ICC.

A Wald test was conducted in order to determine whether iodised salt users differed from non-users in their ability to answer the question items correctly (1 = iodised salt users versus 0 = non-users or not aware of usage). This analysis was also performed for possible gender differences (1 = male versus 0 = female). Differences between the groups are shown as z-values. Statistical analyses were conducted using SPSS version 26.0 and the statistical software R version 4.0.2. The Rasch model analysis was performed with the R package eRm.

## Results

### Characteristics of the study groups

The two study samples did not differ with regard to age and BMI, but the Greek sample included a higher percentage of females than the German group (51% versus 35%). Only 13% of the German and 7% of the Greek sample reported that they had attended a nutrition course in the past, but overall, the majority of the students (Germany: 96%, Greece: 94%) was moderately or very interested in nutrition. In Germany, 17% of the study sample indicated to follow a vegetarian or another type of plant-based (pescetarian or flexitarian) diet and 2% a vegan diet (Table 2).

**Table 2:** Participants´ characteristics

		Germany (*n* = 359)	Greece (*n* = 403)
**Age (in years)**	Mean (± SD)	22.4 (± 3.2)	21.1 (± 2.4)
	Median (IQR)	22.0 (20.0 – 24.0)	21.0 (20.0 – 21.0)
	Not indicated (n [%])	1 (1)	–
**BMI (kg/m**^**2**^**)**	Mean (± SD)	22.6 (± 3.2)	23.4 (± 3.3)
	Median (IQR)	22.0 (20.5 – 24.3)	23.1 (21.2 – 25.1)
	Not indicated (n [%])	11 (3)	–
		n (%)	
**Gender**	Female	126 (35)	204 (51)
	Male	229 (64)	199 (49)
	Not indicated	4 (1)	–
**Attendance of a** **nutrition course**	Yes	45 (13)	26 (7)
	No	314 (87)	377 (93)
**Interest in nutrition**	Not at all	15 (4)	24 (6)
	Moderate	232 (65)	277 (69)
	Very interested	112 (31)	102 (25)
**Dietary habits**	Omnivorous	294 (79)	–
	Flexitarian^a^	12 (3)	–
	Pescetarian^b^	4 (1)	–
	Vegetarian	46 (13)	–
	Vegan	8 (2)	–
	Not indicated	7 (2)	–

*SD* Standard deviation, *IQR* Interquartile range

^a^ self-reported “rare or very rare meat consumption” or self-reported “flexitarian diet”

^b^self-reported “vegetarian diet with the consumption of fish” or self-reported “pescetarian diet”

### Use of iodised salt

In Germany, 41% (*n* = 147) of the participants indicated to use iodised salt (salt with added iodine or with iodine, fluoride and folic acid) at home, while 37% of the Greek participants (*n* = 148) indicated to do so. Thus, the majority of both samples either used a non-iodised type of salt (33% in Germany, 16% in Greece) or were not aware of the type of salt they used at home (16% in Germany, 45% in Greece). In the German sample, 1% and in the Greek sample 2% of the students reported that they did not use salt at all.

**Fig. 1:**
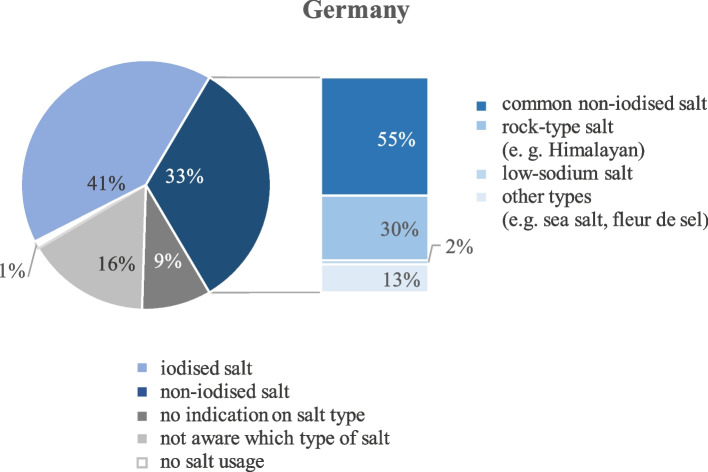
Types of salt used by the study participants in Germany (*n* = 359)

**Fig. 2:**
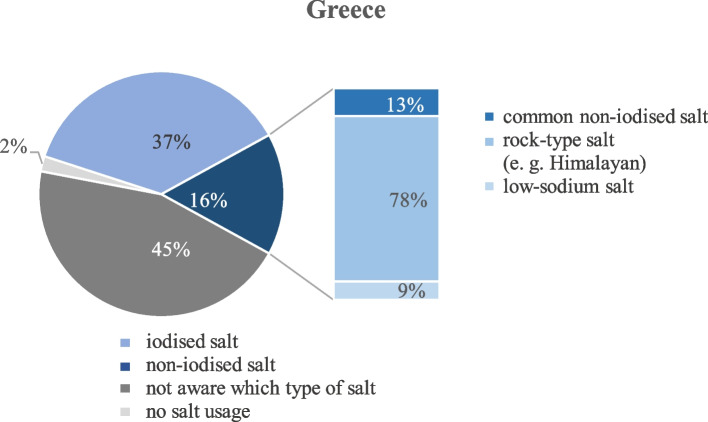
Types of salt used by the study participants in Greece (*n* = 403)

Of those who indicated to use non-iodised salt, 55% and 13% used a common non-iodised table salt, 30% and 78% used a rock type salt like Himalayan salt, 2% and 9% a low-sodium salt (German and Greek sample, respectively), and 13% of the German group indicated to use another type of salt, e.g. sea salt or fleur de sel (Figs. 1 and 2).

The univariate analysis showed no significant differences in age, gender and BMI between participants who used or did not use iodised salt at home in both countries (*p* > 0.05). Multivariate analyses, however, showed that in Germany the iodised salt usage increased with age (AOR = 1.08 [95% CI: 1.001; 1.16]; *p* = 0.048) (Table 3), although this effect was quite weak and only borderline statistically significant.

**Table 3:** Differences between users and non-users* of iodised salt

**Germany (*n* = 322)**	**Use of iodised salt (*n* = 147)**	**No use/no awareness (*n* = 175)**	**Univariate Analysis**		**Multivariate Analysis**	
	**Median** **(IQR)**		**Effect** **size** **(Cohen)**^a^	***p*-value**	**AOR (95%-CI)**^b^	***p*-value**
***Age***^***c***^** (*years*)**	22 (20–25)	21 (20–23)	0.1	0.07	1.08 (1.001; 1.16)	**0.048**
***BMI***^**d**^** (*kg/m***^***2***^**)**	22.2 (20.7–24.5)	21.9 (20.2–24.4)	0.06	0.32	1.046 (0.97; 1.12)	0.25
***Knowledge score (score points)***	14 (12–17)	13 (12–15)	0.13	**0.02**	1.07 (0.98; 1.17)	0.15
	**n (%)**		**OR (95% CI)**^e^	***p*-value**	**AOR (95%-CI)**^b^	***p*-value**
***Gender***^f^						
Male	94 (64)	117 (68)	Ref.		Ref.	
Female	52 (36)	55 (32)	1.18 (0.74; 1.88)	0.49	1.10 (0.64; 1.90)	0.72
***Nutrition course***						
No	119 (81)	164 (94)	Ref.		Ref.	
Yes	28 (19)	11 (6)	3.51 (1.68; 7.30)	** < 0.01**	3.26 (1.55; 6.87)	** < 0.05**
***Interest in nutrition***						
Not at all	5 (3)	9 (5)	Ref.		Ref.	
Moderate	97 (66)	115 (66)	1.52 (0.49; 4.68)	0.47	1.32 (0.42; 4.19)	0.63
Very interested	45 (31)	51 (29)	1.59 (0.50; 5.09)	0.43	1.002 (0.29; 3.38)	0.99
***Attention to food labels***^f^						
Rarely/never	93 (65)	129 (74)	Ref.		Ref.	
Sometimes/always	51 (35)	45 (26)	1.57 (0.97; 2.54)	0.065	1.37 (0.81; 2.32)	0.24
**Greece (*n* = 394)**	**Use of iodised salt (*n* = 148)**	**No use/ no awareness (*n* = 246)**	**Univariate Analysis**		**Multivariate Analysis**	
	**Median (IQR)**		**Effect size (Cohen)**^a^	***p*-value**	**AOR (95%-CI)**^b^	***p*-value**
***Age*** ***(years)***	21 (20–22)	21 (20–21)	0.05	0.33	1.02 (0.93; 1.12)	0.62
***BMI*** ***(kg/m***^***2***^***)***	23.3 (21.2–25.5)	22.9 (21.2–24.9)	0.06	0.25	1.03 (0.97; 1.10)	0.31
***Knowledge*** ***score*** ***(score*** ***points)***	10.5 (9–12)	9.5 (8–12)	0.15	** < 0.01**	1.11 (1.02; 1.20)	** < 0.05**
	**n (%)**		**OR (95% CI)**^e^	***p*-value**	**AOR (95%-CI)**^b^	***p*-value**
***Gender***						
Male	68 (46)	22 (31)	Ref.		Ref.	
Female	80 (54)	50 (69)	1.21 (0.81; 1.83)	0.35	1.26 (0.82; 1.95)	0.29
***Nutrition course***						
No	133 (90)	237 (96)	Ref.		Ref.	
Yes	15 (10)	9 (4)	2.97 (1.26; 6.97)	** < 0.01**	3.22 (1.34; 7.78)	** < 0.01**
***Interest in nutrition***						
Not at all	8 (5)	16 (6)	Ref.		Ref.	
Moderate	105 (71)	169 (69)	1.24 (0.51; 3.00)	0.63	1.18 (0.47; 2.98)	0.72
Very interested	35 (24)	61 (25)	1.15 (0.45; 2.95)	0.77	1.06 (0.39; 2.88)	0.91
***Attention to food labels***						
Rarely/never	109 (74)	156 (63)	Ref.		Ref.	
Sometimes/always	39 (26)	90 (37)	0.62 (0.39; 0.97)	** < 0.05**	0.51 (0.32; 0.82)	** < 0.01**

^*^Participants who were not aware of the salt they used were combined with those who used non-iodised salt

*IQR* Interquartile range, *OR* Odds Ratio, *AOR* Adjusted Odds Ratio, *Ref.* Reference category

^a^Mann-Whitney U-test (effect size according to Cohen)

^b^Logistic regression model (AOR with a 95% confidence interval)

^c^*n* = 321

^d^*n* = 313

^e^Chi-square test on independence (OR with a 95% confidence interval)

^f^*n* = 318

In both Germany and Greece, prior attendance of a nutrition course was positively associated with the use of iodised salt (AOR_Germany_ = 3.26 [95% CI: 1.55; 6.87]; *p* < 0.05 and AOR_Greece_ = 3.22 [95% CI: 1.34; 7.78]; *p* < 0.01). However, the participants’ indicated interest in nutrition had no such effect (Table 3).

About one third of both samples (31% in Germany and 33% in Greece) indicated to regularly pay attention to nutrition information on food labels (data not shown). Only in Greece, this was associated with a less frequent use of iodised salt, whereas in Germany, there was no effect of reading food labels on the use of iodised salt (Table 3).

### Knowledge about iodine/iodised salt

**Fig. 3:**
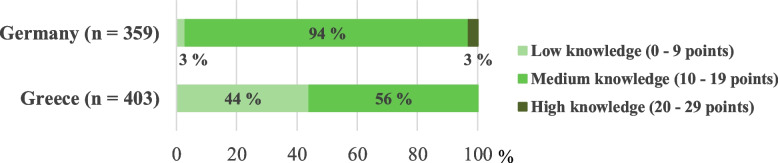
Participants with a low, medium and high knowledge (knowledge score) about iodine/ iodised salt

None of the study participants answered all of the 29 question items correctly. As shown in Fig. 3, the majority of the German sample (94%), but only 56% of the Greek sample had a medium knowledge, whereas almost half of the Greek study group (44%) had a low knowledge. Only of the German participants, 3% reached a high knowledge score.

The median knowledge scores were 14 (IQR: 12—16) and 10 (IQR: 8—12) in Germany and Greece, respectively, and differed significantly between the two countries (*p* < 0.001; data not shown). With an increasing knowledge score, the use of iodised salt also increased significantly in both countries, although the effect size was small (*r* = 0.13 in Germany and *r* = 0.15 in Greece), and after adjustment for possible confounders, the observed effect persisted only in the Greek sample (AO*R* = 1.11 [95% CI: 1.02; 1.20]; *p* < 0.05) (Table 3).

**Fig. 4:**
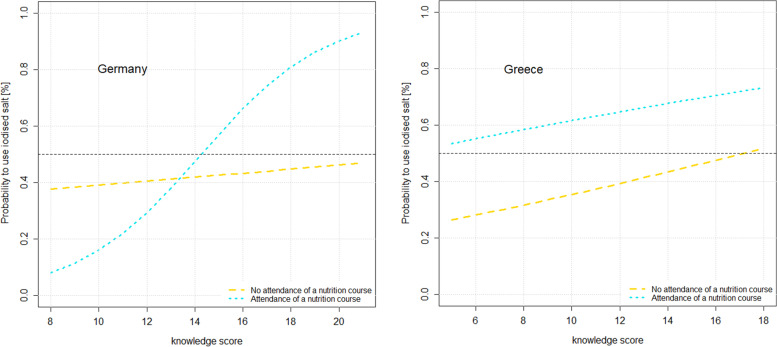
Interaction effects of knowledge and nutrition course attendance on the probability to use iodised salt

Moreover, a test on interaction effects showed that the association between knowledge score and use of iodised salt was modified by the attendance of a nutrition course, but only in the German sample (AOR = 1.46 [95% CI: 1.05; 2.03]; *p* < 0.05). Thus, in this sample, a good knowledge about iodine and salt (knowledge score above 14) and previous attendance of a nutrition course (dotted line, Fig. 4) together had a strong positive effect on the use of iodised salt.

**Fig. 5:**
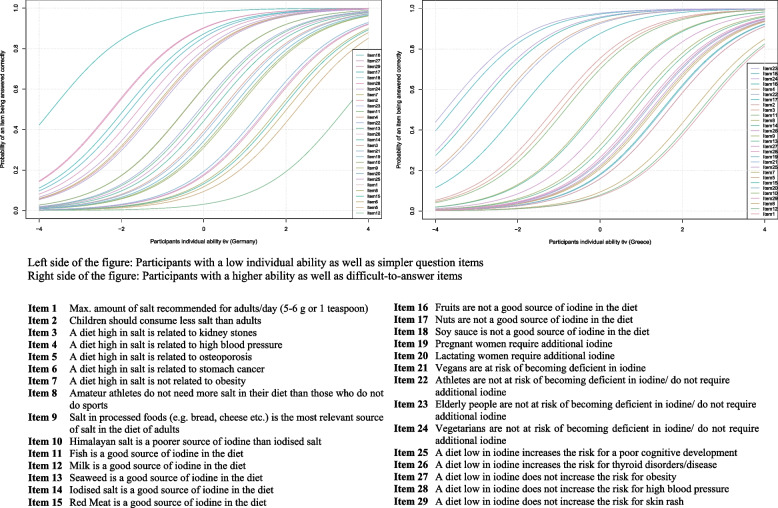
Probability of correct answers taking into account the person´s ability and the difficulty of the question

The results of the Rasch modelling analysis (ICC plot, Fig. 5) show that the probability of a question being answered correctly was higher in students with better knowledge and personal ability. However, there were some differences between Germany and Greece as to the questions/items that were answered correctly most often. While in Germany, question items 16, 17, 18, 27, 28 and 29 (about the facts that fruits, nuts and soy sauce are *not* considered good sources of iodine and that a diet low in iodine is *not* associated with obesity, skin rash or high blood pressure) were most often answered correctly, in the Greek sample, this was the case for question items 4, 16, 17, 18, 22, 23 and 24 (about the fact that a diet high in salt is related to high blood pressure, about foods that are stipulated *not* to be a good iodine source and population groups that are *not* at a higher risk of iodine deficiency, i.e. elderly people, vegetarians and athletes).

Participants in both countries not only lacked knowledge about the fact that iodised salt is considered a good iodine source (item 14), but also about population groups that *are* at risk of iodine deficiency (items 19, 20, 21) and health risks that *are* associated with a diet low in iodine (items 25, 26). Yet, Greek students answered these questions (items 14, 19, 25, 26) correctly more often than German students, and they also had a better knowledge about fish being a good iodine source (item 11). They had, however, less knowledge than German students about seaweed being an iodine source (item 13), about the fact that the specialty salt “Himalayan salt” has a lower iodine content than standard iodised salt (item 10) and about lactating women and vegans being at risk of iodine deficiency (items 20, 21). Surprisingly, participants in both countries rarely identified milk as a relevant iodine source (item 12). In fact, this item was answered least correctly in the German sample and the second least correctly in the Greek sample (Fig. 5).

### Differences between users and non-users of iodised salt regarding their knowledge

The Wald test revealed that users and non-users in both countries differed with regard to their knowledge in different ways: when considering all significant differences, users in the German sample answered more questions correctly than non-users (4 vs. 2 items), whereas this was not the case in the Greek sample (1 vs. 2). However, only considering the iodine-related questions, in the German sample, users were significantly better in answering question items 14, 19 and 20 (thus identifying iodised salt as a good iodine source and the population groups at risk of iodine deficiency), while in the Greek sample, this was the case with regard to item 26 (a diet low in iodine is associated with thyroid disorders). In contrast, the German non-users had a better knowledge about the fact that elderly people do *not* require additional iodine (item 23) and the Greek non-users about vegetarians *no*t requiring additional iodine (item 24) as well as about the fact that nuts are *not* a good iodine source (item 17) (Table 4). There were no significant differences observed with regard to the other question items.

**Table 4:** Differences between users/non-users of iodised salt with regard to their knowledge (Wald test)*

Germany				Greece			
Question item**	**% u/non-u **^**a**^	**z-value**^**b**^	***p*-value**	Question item**	**% u/non-u **^**a**^	**z-value**^**b**^	***p*-value**
**Item 1**	23/9	-3.165	0.002	**Item 17**	68/78	3.321	0.001
**Item 5**	5/11	2.470	0.013	**Item 24**	85/94	3.626	< 0.001
**Item 14**	55/32	- 3.642	< 0.001	**Item 26**	32/19	- 2.487	0.013
**Item 19**	41/25	- 2.475	0.013				
**Item 20**	26/13	- 2.592	0.010				
**Item 23**	65/81	3.829	< 0.001				

^*^only statistically significant differences are shown

^**^for a detailed description of the question items see Table 1

^a^percentage of (conscious) iodised salt users/non users who gave a correct answer

^b^negative z-value means better knowledge in iodised salt users; positive z-value means better knowledge in non-iodised salt users

### Differences between female and male students regarding their knowledge

In both countries, female students were better in answering the questions correctly than the males: significant differences in 6 vs. 2 and 4 vs. 3 items correctly answered in the German and Greek sample, respectively (Table 5).

**Table 5:** Differences between females and males regarding their knowledge (Wald test)*

Germany				Greece			
Question item**	**% f/m**^**a**^	**z-value**^**b**^	***p*-value**	Question item**	**% f/m**^**a**^	**z-value**^**b**^	***p*-value**
**Item 4**	71/52	2.109	0.035	**Item 3**	59/45	2.071	0.038
**Item 5**	14/7	1.981	0.048	**Item 4**	90/79	2.420	0.016
**Item 7**	70/79	- 1.985	0.047	**Item 7**	9/15	- 2.253	0.024
**Item 13**	64/41	2.747	0.006	**Item 17**	66/82	- 4.198	0.000
**Item 20**	26/14	2.248	0.025	**Item 18**	91/96	- 2.370	0.018
**Item 23**	69/76	- 2.022	0.043	**Item 19**	18/9	2.013	0.044
**Item 25**	25/13	2.661	0.008	**Item 26**	32/15	3.678	0.000
**Item 26**	59/38	2.936	0.003				

^*^only statistically significant differences are shown

^**^for a detailed description of the question items see Table 1

^a^percentage of females/males who gave a correct answer

^b^positive z-value means better knowledge in females; negative z-value means better knowledge in males

When considering only the iodine-related questions, they had a significantly better knowledge than males about question items 13, 20, 25 and 26 in Germany (seaweed is a good source of iodine, lactating women are at risk of iodine deficiency and the association of a low iodine intake with poor cognitive development/ thyroid disorders) and about items 19 and 26 in Greece (pregnant women are at risk of iodine deficiency and the association of a low iodine intake with thyroid disorders). Males had a better knowledge about item 23 (elderly people are not at risk of iodine deficiency) in Germany and about items 17 and 18 in Greece (nuts as well as soy sauce not being a good source of iodine) (Table 5). No gender differences were observed with regard to the other question items.

### Differences between students who follow a plant-based or an omnivorous diet in Germany

Vegans in the German study sample identified iodised salt and seaweed as an iodine source more frequently (75% and 88%, respectively) than omnivores (43% and 47%, respectively) or students who followed another type of plant-based diet (37% and 53%, respectively). However, only one quarter (25%) of the vegans was aware of the increased risk for iodine deficiency by following a vegan diet. This was also observed in those who followed a vegetarian or another plant-based diet (23%), but not in omnivores, who had a higher awareness about the fact that vegans are at risk of iodine deficiency (40%).

Despite the fact that 75% of the vegans identified iodised salt as an important iodine source, only 57% of them indicated to use iodised salt at home. Nevertheless, this use level was higher than that observed in the students who followed another type of plant-based diet or were omnivores (36% and 47%, respectively) (Table 6).

**Table 6:** Use of iodised salt and knowledge about iodine depending on the type of diet (Germany)

Type of diet	Use of iodised salt	Knowledge about the fact that…		
		iodised salt is a good iodine source	seaweed is a good iodine source	vegans may have an increased risk of becoming iodine-deficient
	**n (%)**			
**Vegan (*n* = 8)**	4 (57)^a^	6 (75)	7 (88)	2 (25)
**Other plant-based diets: Vegetarian/Flexitarian/Pescetarian (*n* = 62)**	20 (36)^b^	23 (37)	33 (53)	14 (23)
**Omnivorous (*n* = 282)**	123 (47)^c^	122 (43)	132 (47)	114 (40)

^a^*n* = 7

^b^*n* = 55

^c^*n* = 260

## Discussion

The present data analyses were undertaken with the aim to evaluate the knowledge and awareness about iodine and (iodised) salt and the use of iodised salt among university students in Germany and Greece. Our study revealed a relatively low degree of iodised salt usage, with 41% in the German and 37% in the Greek study group. The use level in the German study group was thus only about half of that observed in previous studies (75%—82% in studies from 1996 to 2021) [1, 23–26] and in the Greek sample, it was about twice as high as in 2007, i.e. 37% versus 18% [1]. However, it has to be taken into account that there are limitations when directly comparing these data since one of the previous studies from Germany was conducted in a non-adult population [24] and in some of the studies, data on the iodised salt market segment for household use were collected [1, 25], which are not the same as consumption data. Nevertheless, the iodised salt use level in our study did not reach 90%, as recommended by the WHO and UNICEF [1]. The low prevalence of reported iodised salt usage may partly be explained by the fact that many participants were not aware of the type of salt they were actually using. This was particularly evident in Greece, where 45% of the study group was not aware of the type of salt used at home. Yet, the results of this study also revealed an insufficient knowledge about iodine/iodised salt among the study population, illustrated for example and very surprisingly by the fact that participants in both countries did not often identify iodised salt as a good iodine source, although iodised salt is one of the most important iodine sources in human nutrition. This is in contrast to results of a study from Norway in bachelor students [27], but in agreement with a study in South African adults [28]. Moreover, our participants often correctly identified foods that do *not* serve as good iodine sources, but—in line with results from the Norwegian study [27]—seldom knew that milk makes a very important contribution to the iodine supply. Despite the overall quite poor knowledge about iodine in the Norwegian study, the students´ knowledge about milk being an important source of calcium, which is vital for bone health, was good. However, this did not lead to a frequent milk consumption, as the majority of those students reported to rarely consume milk or having omitted milk from their diet. From our results, it cannot be concluded whether a better knowledge about the relevance of milk/milk products for the iodine supply would actually have lead to an increased iodine intake via milk/milk products consumption.

The participants in our analysis were also more likely to correctly identify health conditions *not* associated with a low iodine intake (Germany) or population groups *not* at an increased risk of iodine deficiency (Greece) than to identify potential negative health consequences of iodine deficiency (poor cognitive development and thyroid disorders) or to indicate population groups that require additional iodine or are at an increased risk of iodine deficiency (pregnant or lactating women and vegans). This was particularly evident for the male students. Female students in both countries, by contrast, had a much better specific knowledge about iodine deficiency and associated health risks and about population groups at risk of iodine deficiency. With regard to foods that *do* serve as good dietary iodine sources, there was only one difference observed between males and females, i.e. that German females knew more often that seaweed is an iodine source. In our previous study [20], which included six countries in Asia and Europe, males—by contrast—had a significantly better knowledge about iodised salt being a good iodine source.

Despite the higher awareness of females about the impact of iodine on health outcomes and about the population groups at risk of iodine deficiency in our study, they did not use iodised salt more often than the males, which is in line with the results of others [29] who also did not observe gender differences in the use of iodised salt. This discrepancy could be caused by the lack of ability to correctly identify good iodine sources and thus act appropriately when choosing foods. Yet, an adequate iodine intake is particularly important for women of childbearing age who may be at risk of an insufficient iodine supply, if they become pregnant.

The overall relatively poor knowledge observed in our study is in agreement with that seen in previous studies from Germany, Italy and the aforementioned study from Norway [26, 27, 29], in which university and even medical students lacked knowledge about iodine-related issues. In the Norwegian study, some students even mentioned that they had never heard of iodine before [27]. It may thus be assumed that the lack of knowledge observed in our study is a general problem in young European adults. Moreover, at least in Germany—based on data from 1996 [26] —, inadequate iodine-related knowledge may either have persisted throughout the past decades or, after an improvement following the introduction of salt iodisation and the assumed greater awareness about iodine-related topics, may have decreased again in the past years. This is of concern since information, communication and education cannot only improve knowledge, but are also important to increase the use of iodised salt [16, 30]. Interventions to increase awareness and knowledge are particularly important in countries with an endemic iodine deficiency, where the use of iodised salt and the consumption of foods rich in iodine is essential for contributing to an adequate iodine intake, especially for women who wish to become or are pregnant or lactating as their iodine status is crucial for fetal development [1].

The inadequate knowledge observed in this study is also worrying in the context of a growing interest in plant-based diets, often among young and highly educated people [31, 32], since strict adherence to a vegan diet could increase the risk of developing iodine deficiency [33]. In our German study sample, 2% of the students indicated to follow a vegan diet, which is a higher proportion than the estimated percentage of vegans in the German population as a whole (between 0.1% and 1%, based on data/reports published in 2007 and 2015) [34].

A food that has a substantial iodine content is seaweed, which is also becoming increasingly popular, especially among vegans, in Western countries [35, 36]. As seaweed can vary greatly in its iodine content, there may be a risk of an excessively high iodine intake with negative health consequences when eating it [37]. Vegans in our study identified seaweed more frequently than other participants (88% vs. 53% of vegetarians/flexitarians/pescetarians and 47% of the omnivores) as a relevant iodine source. However, this study did not allow to assess whether this better knowledge was associated with a more frequent use of seaweed by the vegans, but Eveleigh et al. [37] recently concluded from a systematic review that very high iodine intakes in vegans may be a result of seaweed consumption. Thus, vegans—who do not consume animal products including milk or fish—may on the one hand be at an increased risk of iodine deficiency, but on the other hand also at risk of an iodine excess. Only 25% of our vegan participants knew that their type of diet may pose a risk of iodine deficiency, and this was comparable to the participants who followed another type of plant-based diet (23% of the vegetarians/flexitarians/pescetarians). Moreover, when considering that 75% of the vegans in our study identified iodised salt as a good iodine source, it is surprising that only 57% of them consciously used iodised salt in their households. Nevertheless, this use level was still higher than that observed in those who followed a vegetarian/flexitarian/pescetarian or omnivorous diet, i.e. 36% and 47%, respectively. It has to be acknowledged, however, that the number of vegan participants in our study was low (*n* = 8).

It is important that individuals make food choices and related dietary decisions based on good information and knowledge in order to ensure an adequate nutrient supply.

Despite the rather poor knowledge about iodine and iodised salt both in the German and Greek samples included here, our results suggest that an increasing level of knowledge is associated with a more frequent conscious use of iodised salt, although, after adjustment for multiple factors (age, gender, BMI, attendance of a nutrition course, interest in nutrition, food label use and knowledge score), this was only significant in the Greek sample. The attendance of a nutrition course, however, was associated with a more frequent use of iodised salt in both countries, even after adjustment. Furthermore, in the German sample, the interaction of a higher knowledge (score) and the prior attendance of a nutrition course was linked to an even higher frequency of iodised salt use. This shows that nutrition courses may be important as sound and reliable sources of information and education about nutrition and health. Indeed, in a study from Italy in school children, it was reported that their second most important source of nutritional information about iodine—after media sources like the internet, magazines, radio and television—were presentations on iodine at school [16].

Interestingly, most of our study participants were moderately or very interested in nutrition, but a higher interest in nutrition was not clearly associated with a more frequent use of iodised salt, which may partly be caused by ineffective and unsustainable ways of informing the public about iodine and iodised salt.

Furthermore, paying attention to nutrition information on food labels when buying food did not result in a higher use level of iodised salt in Germany and, unexpectedly, it was associated with an even lower use level in Greece. This could have been caused by reservations and misconceptions about additives and foods produced with iodised salt, since they may be considered less natural and less healthy than ordinary foods [38]. This is also supported by results of a recent survey from Germany, in which the decreasing usage of iodised salt by the food industry was partly explained with a low acceptance of iodised salt by the consumers [39]. However, we cannot deduce the consumption of foods produced with iodised salt from our study.

In some segments of the population, there is a trend towards the consumption of specialty salts like Himalayan salt, sea salt, fleur de sel or others, assuming that these salts are more natural or healthier. However, since they contain less iodine than iodised table salt, they contribute to a lesser extent to an adequate iodine status [40, 41]. This information does not seem to reach consumers, since the participants in our study were not very likely to know that Himalayan salt has a lower iodine content than iodised salt. This is even more remarkable since less than half of the participants in both countries indicated to use iodised salt, and many of the students—especially in the Greek sample—used a rock type salt like Himalayan salt or other salts like sea salt or fleur de sel instead. This could also partly be rooted in a negative attitude towards food additives or foods produced with iodised salt [38–40].

### Strengths and limitations

This study provides preliminary insights into the use of iodised salt and iodine-specific knowledge of university students in Germany and Greece, which is important because there is a lack of data about the household use of iodised salt, especially among young adults. Apart from that, knowledge and the use level of iodised salt were assessed in relation to the nutritional behaviour, at least in the German sample of this study, which also should be examined in further studies. One strength of this study was the use of the same methodological approach including the procedure followed for the recruitment of the students and the same questionnaire in the participating countries, which allowed direct comparisons of the results between the two countries. Concerning the use of the Rasch modeling approach in our study, the advantage of this method was that differences in the participants´ knowledge between Germany and Greece could be easily assessed and visualised. In addition, when making assumptions about more general associations, the advantage of the Rasch model lies in the independence of the results from the sample selected, meaning for instance that the selection of persons from a defined population does not have to be random since the results can be transferred to all persons with a certain ability or knowledge [42]. This is important for the validity of the results revealed from the Rasch model analysis, since the number of participants in our study was rather small. A limitation of our study was the fact that our data were based on self-reported information, thus, our participants´ actual behaviour may have differed from the reported one. Moreover, our results could have been influenced by methodological issues. For instance, since in our study in multiple-choice questions with more than one correct answer, not only the ticking of correct answers, but also the non-ticking of incorrect answers was defined as “correct”, it cannot be concluded with absolute certainty whether the participants gave the correct answers deliberately in every case or not. The problem of possibly not accounting for participants´ guessing in using the so-called number right scoring method—which is widely used in multiple-choice tests—is well-known, but this method has nevertheless been used for decades [43].

A possible solution to minimise the effect of guessing on test reliability is to increase the number of items in the test, as well as the number of alternative choices in multiple choice questions [43], which was done in our study. Yet, despite the uncertainties regarding the use of multiple choice questions, there is also uncertainty as to whether we made a valid classification of the participants in terms of their knowledge and have therefore measured their “true” level of knowledge. Furthermore, our study groups consisted only of university students in Berlin, Larisa and Thessaloniki and were thus neither representative of the student populations in Germany and Greece nor for the general populations in these countries. Therefore, in order to gain more representative and thus reliable results about the knowledge and awareness regarding iodine and iodised salt and the actual iodised salt usage at population level, it is desirable that representative data be collected in future studies.

## Conclusions

The present study shows a relatively low level of conscious iodised salt use among university students in Germany and Greece. This may partly be explained with inadequate knowledge about the role of iodine/iodised salt in human nutrition and health. In addition, almost half of the participants in Greece were not able to recall the type of salt used at home, suggesting that a lack of awareness could have been another cause for the low use level observed.

Considering the overall rather unsatisfactory knowledge, it is emphasized that education on the importance of iodine should be offered consistently and continuously to obtain a sustainable effect. As the attendance of nutrition courses and a better knowledge about iodine/iodised salt had a positive impact on the use of iodised salt, it would be desirable that education about iodine be offered in nutrition courses in school curricula to ensure equal access to this information for all levels of education, preferably starting from an early age. There is also a need for widespread information campaigns on the importance of iodine in order to assure a sustainable awareness about iodine and related health matters in the public across all age groups, health care professionals, food producing companies and wide-reaching multipliers on social media platforms, as an information source particularly for younger people. In order to be effective, these information campaigns should be planned in a target-group specific manner.

A better knowledge about the importance of iodine and iodised salt for health could not only have a positive impact on the use of iodised salt in private households, but also improve consumer acceptance and demand for processed foods manufactured with iodised salt (such as bread, cheese or meat), thus contributing to an increased iodised salt usage in food manufacturing, which would eventually contribute to an improved iodine status of the population, including those at risk of iodine deficiency.

## Data Availability

The datasets used and/or analysed during the current study are available from the corresponding author on reasonable request.

## Abbreviations

AI: Adequate Intake
AOR: Adjusted Odds Ratio
BMI: Body Mass Index
CI: Confidence Interval
EFSA: European Food Safety Authority
ICC: Item Characteristic Curve
IDD: Iodine Deficiency Disorders
IQR: Interquartile Range
NVS II: Nationale Verzehrsstudie II, national food consumption survey II
OR: Odds Ratio
SD: Standard Deviation
UNICEF: United Nations Children´s Fund
USI: Universal Salt Iodisation
WHO: World Health Organization
